# Supplementary material for: Getting More Out of Biomedical Documents with GATE's Full Lifecycle Open Source Text Analytics
Source: PLoS Comput Biol. 2013 Feb 7;9(2):e1002854. doi: 10.1371/journal.pcbi.1002854 (PMC3567135; doi:10.1371/journal.pcbi.1002854)
Supplement: Dataset S1 — GATE software. Dataset S1 bundled with this paper contains a distribution of GATE (or see http://gate.ac.uk/download/). (TGZ) [file pcbi.1002854.s001.tgz › plos-gate/doc/proposal.html]

GATE 2 original proposal


 **GATE 2: Software Infrastructure for NLP

## Hamish Cunningham, Yorick Wilks, March 1999** 

 **High-level Summary** 

The fundamental aim of this project is to boost productivity in natural language processing (NLP) research in the UK. At present a large amount of time is wasted by highly-skilled researchers who must continually reinvent the wheel when starting a new project or a new set of experiments. As in other fields, language processing work involves repetitive tasks that provide necessary infrastructure but do not directly advance our R&D. Unfortunately, whereas in other fields advanced computational tools are available that fulfil infrastructural needs (e.g. MatLab or Mathematica for mathematicians), no such general infrastructure is available in NLP.

Every PhD thesis or research grant from EPSRC (or the EC) typically begins its software development phase by defining a new set of tools and data structures for tasks such as:

- the management of the textual data to be processed;

- the storage and exchange of information about texts;

- the visualisation of the data (essential for efficient development and debugging);

- the comparison of the results of different configurations of software or with human-produced results;

- accessing resources such as lexicons and dictionaries that each has its own idiosyncratic format.

All of these tasks require robust, engineered software solutions, and all of them are key factors in the success of UK NLP research. The types of data structure typically produced and manipulated by NLP programs are large and complex, and without good tools to manage storage of, and allow succinct graphical viewing of, these structures, we work below our potential. At this stage in the progress of our field, no one should really have to write a tree viewing program for the output of a syntax analyser, for example, or even have to do significant work to get an existing viewing tool to process their data. While it is true that a number of specialised infrastructures exist, these are tied into particular theoretical commitments, for example attribute-value matrices evaluated under unification. Particularly at a time when statistical and hybrid statistical/symbolic work is so prevalent, these specialised systems cannot fulfil the role of a general NLP architecture.

In addition, many common language processing tasks have been solved to an acceptable degree by previous work and should be reused. Instead of writing a new part of speech tagger, or sentence splitter, or list of common nominal compounds, we should have available a store of reusable tools and data that can be plugged into our new systems with minimal effort. Such reuse is much less common than it should be, often because of installation and integration problems that have to be solved afresh in each case, and this is a major waste of research funding.

Building on previous work funded by the EPSRC, this project will eliminate the need for the majority of language processing workers to bother with infrastructure. We will provide the equivalent of Mathematica for language research and development. We will also make available a wide range of building blocks for common tasks (e.g. tagging) and to access common data resources (e.g. WordNet). The infrastructure and the building blocks will be WWW-enabled, and may reside on more than one machine as needed. A server in Sheffield will provide a high quality gateway to architectural services, language engineering building blocks and data resources.

Our aim, then, is to advance UK research in language processing, and enable us to preserve our leading role in the EC�s programmes, particularly at a time when developments on the Internet are driving investment in this area and have led to a number of new commercial developments. Sadly, a high proportion of the new companies and products appearing now are based in the US. Our work will contribute to easing the commercialisation of language technologies by providing an efficient, robust and well-documented data management substrate that can be delivered as part of applications (without any of the graphical development tools that a researcher needs). One advantage that we have over the US is our proximity to multi-lingual continental Europe: our infrastructure is both language-neutral and ideally suited to minimising integration problems in multi-site projects.

Within our own research group at Sheffield, the development proposed here is intended to underlie and enable a rolling experimental programme of research in Information Extraction (where we have just attained the highest score for co-reference resolution in the MUC7 DARPA competition � see

www.muc.saic.com), multi-lingual text content access and summarisation, dialogue processing, word-sense disambiguation and extension, lexical storage and belief/knowledge representation.

Last but not least, academics teaching computational linguistics, natural language processing and natural language engineering, and some parts of cognitive science, linguistics and modern languages can all benefit from easy-to-use graphical tools for manipulating language and language-related data structures.

Below we give comments on reviews of a previous version of this proposal, then section 1 describes our previous work in this area, and introduces the key personnel involved. Section 2 gives extensive technical detail of the work we will carry out in the project, beginning with a review of related previous work in the field (2.1) which motivates the broad stroke of our design (2.2), which is broken into three work packages. The technologies we plan to use are described (2.3). Section 3 gives planning details of the work programme, and section 4 additional relevant information for management and results exploitation. Section 5 gives references, and finally there is a Gantt chart of project packages and milestones.

 **Responses to Previous Reviewers� Comments** 

A previous version of this proposal had largely positive reviews. We respond here to negative points that were raised.

The previous proposal included substantial additional research work in Information Extraction (IE) as part of our ongoing programme in that field. This was consistent with our previous EPSRC project, but was thought inadvisable by the review panel since it made the proposal too large and too obviously separable into architecture and IE parts. We have removed that element of the work in this version, and concentrated on the architecture alone.

One reviewer questioned the viability of completing all the work specified within the time limit, using the labour requested. There are two answers to this. First, we have designed the work programme with multiple deliverables and many milestone points that represent real advances. Should we fail to complete every single part of the programme we would still have made a major contribution to the field. Secondly, we have recently completed a similar-sized project (EPSRC GR/K25267), where we have completed or exceeded the original aims of the proposal, on time and within budget. Additionally, that project began with untrained junior staff, several of whom now, after 3 years involvement with the technology underlying this proposal, form one of the most highly skilled Language Engineering development teams. We feel that there is no question that we can complete the work we propose.

It was felt that we should provide more evidence of the uses to which GATE is being put � we have added to section 2.1.4 accordingly.

Doubts were expressed as to whether we have the necessary expertise for experiments on architectural support for speech processing. Dr. Steve Renals [

RMB94, RHR96] of our Department has agreed to act as an advisor to the project in the areas of architectural requirements for supporting integrated speech and text-based NLP research. He has extensive, published, research experience on the Cambridge Abbott speech project and is a member of the EPSRC College of Computing. The NLP and Speech groups at Sheffield already collaborate extensively (e.g. on using IE for tuning language models of unknown words by identifying typical context of common unknown categories, such as names) and this proposal will continue and extend the collaboration.

One reviewer wrote that 1) the work has no scientific basis; 2) asked why not use Java?; 3) asked how will we integrate with Office environments?; 4) said it may become obsolete quickly because commercial software moves so fast; 5) said it will never run fast enough to be useful in industry; 6) said too little time has elapsed to evaluate the success of the first version; 7) said that the proposal has far too much labour allocated. Taking these in turn:

 *- Not science.* The Council has a responsibility to Engineering and Science, and what we do, like all speech and language R&D, is often more the former than the latter. The role of this architecture has been, and will be, to *enable* substantial amounts of evaluable research (and in that sense scientific) at Sheffield and elsewhere, work it will be far harder to do without such underpinning. We offer a leap forward in such UK-based work. As further evidence of this, we are at present collecting code reuse figures for projects using GATE 1, which we will publish in scientific journals (as we have all our other results, whose referees thought they had merit).
 *- Java?* We already support Java (from version 1.5), and have based our design on many of the facilities available on that platform.
 *- Office integration.* We have planned for this, but space limitations and the belief that technical details were less interesting than the design squeezed it out of the original proposal. Please see the new section *Technological Basis*  (2.3). 
 *- Obsolescence.* Cunningham, the designated lead RA, has spent the last 6 years developing in C++, Tcl/Tk and Java in academia, and several years before that developing in COBOL in industry, and his experience suggests that in many cases academic software moves more quickly than commercial software. In any case, we are developing tools whose utility does not depend on the vagaries of current software fashion, but rather address research into fundamental questions of modelling human faculties using computation.
 *- Speed.* From the point of view of the developer, graphical RAD tools are always more resource hungry than applications for the end user (using MS Visual C++ on a 133Mhz Pentium with 32Mb RAM gives an example of slow-running commercial software!). From the point of view of delivering software based on the underlying data management substrate of the architecture, we fail to see how the design we have put forward (that uses conventional database software and the optimised data structures of the TIPSTER architecture committee, which included substantial commercial interests) is likely to be slow, and our reviewer gave no evidence to back up the assertion. 
 *- Too little evaluation time.*  It is now 18 months since the release of GATE 1.0, and we have included details of its take up and use. As noted under point 4) software moves quickly: 18 months ago Java was a little-known novelty language!
 *- Too much labour.* Another reviewer thought the programme *too ambitious* given the labour requested � see above. GATE 1 and the IE components delivered with it ran to 100,000 lines of code in 5 programming languages. Some industrial software process planning literature assumes 10 lines of fully debugged and documented code per programmer per day, which would have meant 27 person years to produce our system (which was in fact produced, under EPSRC funding, along with numerous academic papers and competing in two MUC evaluations etc., in 9 person-years).
 

1. Previous Research and Track Record

The work carried out under EPSRC grant GR/K25267 has led to the development of GATE, a General Architecture for Text Engineering [

CGW95, CWG96a, CWG96b, CWG96c, CHGW96, CHGW97b, CHGW97a, GCW96, RGHC97] and an advanced 'Vanilla' Information Extraction system (VIE) [GWH95,  GH96, GR97a,  GH97a, WGW96, TWY96, AHG97, GW97]; these have been distributed to more than 80 sites in the UK and elsewhere. At the time of writing GATE is under active consideration as an architecture for distribution within the US DARPA community, a development that would bring substantial prestige (though not reward) to UK Language Engineering. The success of the work indicates the possibility of developing a truly generic applications delivery environment for language processing research and development, within the framework of a world-class research programme in Information Extraction (IE). Note 1: if you prefer a hyp
 **1.1 Previous Research: GATE / VIE 1** 

Version 1.0.0 of GATE was officially released in October 1996 and user experience has proved both the demand for such a system and its practicality.  There is now a real possibility for GATE to become a sound basis for work in the whole field of computing with human language (see

part 2). The final version 1 release of GATE (1.5, Spring 1998): 


- facilitates reuse of NLP components by reducing the overheads of integration, documentation and data visualisation;

- facilitates multi-site collaboration on IE research by providing a modular base-line system (VIE) with which others can experiment;

- facilitates comparative evaluation of different methods by making it easy to interchange modules;

- facilitates task-based evaluation, both of "internal'' components such as taggers and parsers, and of whole systems, e.g. by using materials from the ARPA

MUC programme [GS96] (whose scoring software is available in GATE, as is the Parseval tree scoring tool [Har91], and a generic annotation scoring tool [GR97b]); 

- contributes to increased software-level robustness, quality and efficiency in NLP systems, and to their cross-platform portability (all UNIX systems, Windows NT and Windows 95; native support for Java, C++ and Tcl);

- contributes to the portability of NLP systems across problem domains by providing a markup tool for generating training data for example-based learning (it can also take input from the Alembic tool [

DAH97] for this purpose); 


- unifies the two best approaches to managing information about text by combining a

TIPSTER-style database with SGML input/output filters (developed using tools from Edinburgh's Language Technology Group [MBT97]).

GATE has now been installed at over 80 sites in the UK, EU and US, and has, in effect, its own user community, promoting sharing and comparison of LE modular software.  At the time of writing it is under active consideration for distribution within the US DARPA community.  A list of user sites can be found at

http://www.dcs.shef.ac.uk/research/groups/nlp/gate/users.html.

VIE is the freely distributed counterpart of Sheffield's internal information extraction research system LaSIE (Large-Scale IE). Work on LaSIE has led to:

- impressive results in the MUC-6/7 evaluations [

GS96,GWH95] and in the MET Japanese entity recognition task [TWY96] (in both MUC-6 and MUC-7 Sheffield served on the DARPA programme committee); 

- evaluation of a novel approach to Named Entity recognition [

WGW96]; 

- novel and high-performance co-reference resolution algorithms [

GH96a,GH96b,GH97a] (with the highest score in MUC-7); 

- detailed evaluation of the use of semantic networks for IE [GH97b];

- organisation (by invitation) of a seminar on industrial applications of IE [

WG97];

- development of two complementary approaches to IE, based on parsing and pattern-matching respectively.

LaSIE is now being upgraded to process multiple languages and investigate the use of an interlingual domain model to minimise cross-language porting costs by maximising the extent to which system resources are shareable across languages [

AHG97, Kam97], and is forming the basis of several industrially-sponsored projects and customisations for individual companies (see external support list form). In addition the named-entity recognition modules are being used for experiments modelling out-of vocabulary items for speech recognition. 
 **1.2 Track Record** 

The Natural Language Processing group in the Computer Science Department (and ILASH: the Institute for Language, Speech and Hearing) is a major UK group in the field led by Yorick Wilks, who set it up on his return to the UK in 1993, and who will manage this project.  Major NLP R&D efforts in the recent past include:

 **I** ) The LaSIE/VIE IE system that was part of previous research to this proposal (GR/K25267) (see references above) has competed within the US ARPA TIPSTER and MUC competitions and emerged in 1995 as the best IE system outside the US at such tasks as locating proper names, events and descriptions in texts, and determining co-references between them.  Our group has extensive associated work in grammar induction [KGW94] (GR/K66215): the derivation of grammar rules from corpora, which has provided the basis for grammars for LaSIE, and the stimulus for more general work on learning algorithms for adapting NLP (and particularly IE) systems to users.  Within the EC ECRAN project we have developed lexical tuning algorithms to adapt lexicons for IE to new domains, and a corpus based method for deriving verb patterns from machine readable dictionaries to form the basis of another, pattern rather than grammar based, IE system within GATE (this system shares many modules with LaSIE, a form of complementary and competing development only possible within an architecture like GATE).  We hope this year to test, independently and for the first time, the Levin-Dorr hypothesis on the relation of syntactic and semantic distribution of verbs.  Recent experimental work has included a project on a general sense-tagger (against lexicons) [Wil96b], using a number of modules based on different information sources: this work is currently yielding, at 90% of all words correctly sense-resolved, the best published results world-wide on general (all-word) sense resolution, and forms a GATE module contributing to the pattern-based IE project.  The IE technology has also provided the base for the major EC project AVENTINUS on drugs and security [Thu97], is about to be applied in PASTA, a BBSRC-EPSRC bioinformatics project (50/BIF08754) on automatic extraction of

II) Other research has contributed to modules within GATE that (like the lexical tuning mentioned above) aim to make IE more flexible and adapted to new users and domains and to combine the benefits of IE with machine translation and information access via speech and other modalities.  This includes work on advanced models of users in terms of their beliefs, goals, and intentions (the ViewGen model: [

BW91], [WBB91]**,** [WBW91]**,** [Lee95]**,** [LW96b], [LW96a], [WB96] is a dialogue understanding system which reasons about the attitudes of other agents in nested belief structures by ascription - i.e.  assuming that attitudes held in one attitude environment can be ascribed to others).  Such research on adaptive interfaces for IE also includes work on natural human-machine dialogue, by means of a dialogue grammar derived from the British National Corpus.  This work is at an early stage, but the CONVERSE system (to which we contributed) was good enough to win the 1997 Loebner competition in New York [LCB+97] for the most plausible human-machine dialogue partner. 
 **III** ) Automatic lexicon construction: the CRL Lexical Knowledge Base (LKB) was derived as part of a US National Science Foundation grant (of which Wilks was principal investigator) from the LDOCE dictionary and the Collins Spanish-English bilingual dictionary. This resource was perhaps the first automatically derived lexical resource to have extensive practical application, and has been used by various groups (NYU, Brandeis, USC/ISI, CMU/CMT, the US Department of Defense, etc.) to extract information to analyse texts, within the PANGLOSS and TIPSTER ARPA projects.  The group is now a major contributor to the structural lexical resource projects EuroWordnet and PAROLE/SIMPLE funded by the EC. Wilks has a long history of collaboration with major publishers involved in the production of linguistic resources; particularly Longmans (where he has been on their linguistic committee for many years) CUP, Collins and OUP.  In New Mexico he set up the NSF-funded Consortium for Lexical Research. 

The NLP/ILASH group hosted the AISB conference in 1994, and an EPSRC-DTI supported workshop on architectures for NLP in 1996, and an EPSRC-supported workshop on NLP evaluation in 1997.  It has world-wide affiliations, including official collaborations in the area with NEC and Hitachi Laboratories, and is the WWW host for international computational linguistics (ICCL-COLING) and the new UK Computational Linguistics initiative (CLUK).

 **Yorick Wilks**  has been, since 1993, Professor of Computer Science at the University of Sheffield and Director of ILASH, the Institute of Language, Speech and Hearing.  He received his doctorate from Cambridge University in 1968 for work on computer programs that process written English texts in terms of a theory later called "preference semantics": the claim that language is to be understood by means of a search for semantic patterns or "gists" in texts, combined with a coherence function over such structures that minimises effort in the analyser, and can make hypotheses about possible new senses of text words in context. He was a researcher at Stanford AI Laboratory, and then Professor of Computer Science and Linguistics at the University of Essex in England, before founding the Computing Research Laboratory in New Mexico, where he proposed and managed a range of large research projects in NLP, including information extraction (TIPSTER), lexicon development and belief and knowledge structures. He currently leads four EC and two EPSRC projects.  He has published numerous articles and six books in that area of artificial intelligence, of which the most recent are Artificial Believers (with Afzal Ballim) from Lawrence Erlbaum Associates (1991) and Electric Words: dictionaries, computers and meanings (with Brian Slator and Louise Guthrie) from MIT Press, (1996).  He is  on the EPSRC College of Computing, a Fellow of the American Association for Artificial Intelligence, on the boards of some fifteen AI-related journals, and on the International Committee on Computation Linguistics, the management group of CLUK, the SALT2 Committee and Longman's Linglex Committee. 
 **Robert Gaizauskas**  is a lecturer in the Computer Science Department, University of Sheffield and has a DPhil from the University of Sussex in the area of computational logic.  He worked on the design and implementation of several information extraction systems, as well as the EPSRC-supported GATE architecture for text engineering with Wilks.  He is currently investigator on a BBSRC/EPSRC bioinformatics project to construct automatically a structured database of protein active site data from electronic journals in the field of molecular biology, is leading the project with Glaxo-Wellcome and Elsevier Science described above, and is involved in several European-funded projects to do with language engineering and evaluation of language technology.  He has published numerous papers, has acted as a project reviewer and rapporteur for the EC Language Engineering programme, has been involved in the EC Expert Advisory Group on Language Engineering Standards (EAGLES), is on the UK SALT steering committee, and has worked in industry for Fulcrum, a leading information retrieval company. 
 **Hamish Cunningham**  (proposed Senior RA) has an MSc in Computation (UMIST) and has worked as a programmer, systems administrator and (for the last 6 years) researcher in language engineering. He was lead developer of GATE version 1, wrote parts of the Sheffield MUC-6 entry, and also works on example-based IE. He supervised Sheffield's IE team on the AVENTINUS EC LE project.
 **2. Proposed Research in Software Architecture for NLP R&D** 

Progress in natural language engineering (LE) can benefit significantly from a generally acceptable and extensible "architecture": a software infrastructure with which researchers and developers can share, reuse and compare LE modules and resources. The success of the   US DARPA LE programmme has been evidence of this, and our GATE system has both benefited from, and contributed to that movement.  It has helped keep the UK at the forefront of LE world-wide.  Combinations of modules within such an architecture can perform a great range of research and industrial LE tasks.

An illustrative set of the sort of major "use cases" we expect for GATE2 would be: a dictionary publisher layers our common lexical resources model on top of their dictionary and makes it available on the WWW for limited queries with a view to encouraging researchers and generating extra sales of the unlimited version; the TIPSTER organisation (or an EU equivalent) makes its common module pool available for running remotely on their server, processing texts in databases on client machines; a supplier of information retrieval software (such as a large UK finance house) uses the interface to develop a name recognition component and uses the document manager with interface stripped to deliver the component; academics teaching language processing or language learning use the graphical facilities and rapid prototyping capabilities to structure their classes; researchers in etymology use it to collect statistics on word evolution from online newspapers, without copying all the web pages; an EC R&D project uses it as the backbone for developing term substitution and name transliteration add-ins for the Han character word-processing market.

An *architecture* in this proposal is a macro-level organisational pattern for the components and data resources that make up a language processing system; development environments add graphical tools to access the services provided by the architecture.  The GATE architecture does this using three subsystems: (1) GDM, the GATE Document Manager; (2) GGI, the GATE Graphical Interface; (3) CREOLE, a Collection of REusable Objects for Language Engineering. GDM manages the information about texts produced and consumed by NLP processes; GGI provides visual access to this data and manages control flow; CREOLE is the set of resources so far integrated with GATE.

 **2.1 Previous Work** 

Existing systems that provide software infrastructure for NLP can be classified as belonging to one of three types according to the way they manage information about texts:

 **- additive, or markup-based:** information produced is added to the text in the form of markup, e.g. SGML; 
 **- referential, or annotation-based:** information is stored separately with references back to the original text, e.g. the TIPSTER architecture; 
 **- abstraction-based:** the original text is preserved in processing only as part of an integrated data structure that represents information about the text in a uniform, theoretically-motivated, model, e.g. attribute-value structures in the ALEP system.
 **Additive architectures**  for managing information about text add markup to the original text at each successive phase of processing. An architecture based on SGML has been developed at the University of Edinburgh called LT-NSL [TM96]. Tools in a LT-NSL system communicate via interfaces specified as SGML document type definitions (DTDs � essentially tag set descriptions), using character streams on pipe. To obviate the need to deal with some difficult types of SGML (e.g. minimised markup) texts are converted to a normal form before processing. 

The ARPA-sponsored TIPSTER programme in the US, now in its third phase, has also produced a data-driven architecture for NLP systems [

Gri96]. Whereas in LT-NSL all information about a text is encoded in SGML, which is added by the modules, in TIPSTER a text remains unchanged while information is stored in a separate database: the **referential approach**. Information is stored in the database in the form of *annotations*, which associate arbitrary information (*attributes*), with portions of documents (identified by sets of start/end byte offsets or *spans*). Attributes may be the result of linguistic analysis, e.g. POS tags or textual unit type. In this way the information built up about a text by NLP modules is kept separate from the texts themselves. 

The **abstraction-based** approach to managing information about texts is primarily motivated by theories about the nature of the information represented.  ALEP [

Sch94,Sim94], while in principle open, is primarily an advanced system for developing and manipulating feature structure knowledge-bases under unification. 

 **2.1.1 The GATE Document Manager (GDM)** 

Both the additive and referential approaches are serious candidates for the underlying architecture of an optimal infrastructure; [

CHGW97b] compares them in detail: both are  available as standards (SGML; TIPSTER architecture) and are theory-neutral. TIPSTER has a better time/space profile, can take advantage of database technology straightforwardly, is better for random access to data (SGML is inherently sequential [MBT97]), and can represent graph-structured and non-contiguous text structures easily. For these reasons we believe that there are significant advantages to the TIPSTER model and we have chosen this for the core of the GATE Document Manager, with support for import and export of SGML using the Edinburgh tools.

 **2.1.2 The GATE graphical interface (GGI)**  

One of the key benefits of adopting an explicit architecture for data management is that it becomes straightforward to add graphical interface access to architectural services and data visualisation tools, and such a layer is our second pillar: GGI, the GATE graphical interface. GGI has functions for creating, viewing and editing collections of documents which are managed by the GDM and that form the corpora which LE modules and systems in GATE use as input data. It also has facilities to display the results of module or system execution, i.e. new or changed annotations associated with the document. These annotations can be viewed either in raw form, using a generic annotation viewer, or in an annotation-specific way, if special annotation viewers are available. For example, named entity annotations which identify and classify proper names (e.g. organisation names, person names, location names) are shown by colour-coded highlighting of relevant words; phrase structure annotations are shown by graphical presentation of parse trees; coreference chains are shown through highlighting linked phrases.

 **2.1.3 A developing Collection of REusable Objects for Language Engineering (CREOLE)**  

The third pillar of the system is the one that does all the real work of processing texts and extracting information about their content: CREOLE , a Collection of REusable Objects for Language Engineering. CREOLE is not strictly part of GATE at all, but is the set of resources currently integrated with the system. Members of this set currently include:  the VIE English IE components (tokeniser and text structure analysers; sentence splitter; two POS taggers; morphological analyser; chart parser; name matcher; discourse interpreter); the Brill tagger; the

Alvey Natural Language Tools morphological analyser and parser; the Plink parser; the Parseval tree comparison software [Har91]; the MUC scoring tools [GS96]; French parsing and morphological analysis tools from Fribourg and INRIA; Italian corpus analysis tools from Rome Tor Vergata; a wide range of Swedish language processing tools. Other modules to be integrated in the near future include: the ALICE parser from UMIST; the Claws tagger from Lancaster; the neural net parser from Hertfordshire; the Cambridge/CMU statistical language modelling toolkit. 

The process of integrating modules into GATE has been automated to a large degree and can be driven from the interface. The developer is required to produce some C++, Tcl or Java code that uses the GDM TIPSTER API to get information from the database and write back results. The underlying module can be in C/C++, Java or Tcl, or be an external executable written in any language (the current CREOLE set includes Prolog, Lisp and Perl programs, for example).

 **2.1.4 Users of GATE 1**  

We maintain a database of the groups and individuals who are using GATE for a wide variety of tasks (and the feedback we have received from these users has been incorporated in the content of this proposal), for example:

- Teaching undergraduates and postgraduates. Our colleagues in the Universities of Edinburgh, Manchester (UMIST) and Sussex have reported using the system for teaching; the University of Stuttgart has produced a tutorial in German for the same purposes.

- Information Extraction in English, German, French, Spanish and Greek. Our colleagues in Fribourg University collaborated with us on a French IE system; GMS in Germany are working on an IE system, both ILSP and NKSR Demokritus in Athens are developing in Greek, and Incyta in Barcelona are working on Spanish.

- Integrating information extraction with Information Retrieval. The Naval Office of R&D (NRaD) in San Diego is using GATE for research on text summarisation and IE/IR integration and now (April 1998) proposes to use GATE as the basis of their main final demonstration of the whole DARPA TIPSTER project integrating large-scale IE and information retrieval. This is the most substantial accolade yet awarded to GATE.

- Integrating a national collection of NLP tools (for Swedish). See

http://www.sics.se/humle/projects/svensk/svensk.html 

- Converting an MT system to the TIPSTER architecture (the Corelli project at NMSU).

- ESTEAM Inc., of Gothenburg and Athens are using the system for adding name recognition to their MT systems (for 26 language pairs) to improve performance on unknowns.

- The Speech and Hearing group at Sheffield are modelling out-of-vocabulary language using VIE and GATE [

Got98].

- Numerous postgraduates in locations as diverse as Israel, Copenhagen and Surrey are using the system to avoid having to write simple things like sentence splitters from scratch, and to enable visualisation and management of data.

For more details of users and their activities see

http://www.dcs.shef.ac.uk/research/groups/nlp/gate/users.html


 **2.2 The next stage: GATE 2** 

Our proposal is to extend CREOLE to cover as many core areas of language engineering R&D as possible. In support of this aim, GDM and GGI will be developed in a number of significant ways, and a new subsystem, the GATE Resource Manager (GRM), added to extend control regime options and cater for integration of both algorithmic and data resources. Like other software, NLP components comprise both algorithms and data, and are  predominantly one or the other:  for example, a part-of-speech tagger or a parser is best distinguished by its algorithms (e.g. finite state transduction, or feature structure unification over charts); a lexicon or grammar is best described by reference to the data types and tokens it contains (rather than whatever methods are used to access it). GATE  1 was biased towards algorithmic resources, partly because reuse of such resources has been very low in the field, whereas reuse of data is more frequent [

Cun94] (e.g. compare how many new parsers still get written each year, versus how many people use the Penn Treebank). GATE  2 will correct this imbalance, and provide an entirely new way of distributing and accessing NLP data resources.

 **WP 1: The GATE2 core

WP 1.1: A distributed Unicode document manager: GDM 2** 

The second version of the GATE document manager will support a very wide range of human languages by adoption of the Unicode standard, and enable users to select from a variety of SQL databases (via the

JDBC standard) and object-oriented databases, including a free version using the ObjectDesign PSE system. We will extend the current facilities for reading in SGML documents and converting their markup into TIPSTER format to a range of text types including email and RTF, and track the emerging XML standard. Documents and their components will support conversion to HTML for viewing and exporting data separately from the graphical interface. To support IR work we will implement the TIPSTER IR classes, and provide efficient lightweight document management to fit the computationally intensive processes used in this field. Finally, we will support multi-media documents by moving to a transparent representation of document content with variable indexing for different components (text, graphics, pictures, video, audio). Distributed document databases will be supported using the NMSU Computing Research Laboratory�s Java RMI document manager, the supply of which is already agreed.

 **WP 1.2: A standards-based interface: GGI 2** 

GATE 2 will take advantage of the prevalence of object-centred graphical interfaces to provide a look-and-feel that follows de-facto standards and hence has a low learning overhead. For example, there will be object-specific management tools for all domain objects (systems, modules, collections, documents, annotations). These tools will provide tabbed views, drag and drop, flexible customisation (e.g., user-defined filters on annotations will be displayed in separate tabs). In general, the aim is to make GATE look like a desktop incorporating all these management tools, so that the user can easily manipulate systems and documents and also inspect the results associated with each document. An easy-to-learn, easy-to-use interface will increase the willingness of researchers to adopt GATE as a tool and will promote its use as a teaching tool (several users are already using GATE for teaching and there has been considerable interest expressed by colleagues at Sussex, Edinburgh and Lancaster in extending its utility for teaching). The interface will support Unicode, and will allow editable documents with automatic annotation offset recalculation (via a gap buffer). We will investigate the problem of chaining updates to annotation structures during editing. GGI will be a set of Java Beans, so developers will be able to build their own customised interfaces (see 2.1.3).

 **WP 1.3: Agent-based language processing and a new approach to linguistic resource distribution: GRM** 

This new subsystem will:

- encapsulate a range of alternative control regimes, e.g. for distributed operation of cooperating autonomous agents;

- consolidate the algorithmic resource integration mechanisms that currently live in GGI and GDM and extend those mechanisms to support distributed tools;

- add integration and management mechanisms for linguistic resources such as lexicons.

At present, services such as the Lexical Data Consortium, the European Language Resources Association, and the Stuttgart Registry are static servers that require the user to download source code or data and install it on their own machines. We will develop an **Active CREOLE Server** with which researchers can assemble systems from components that may be running on a mixture of their own site, the server site (Sheffield) and at others' sites. The overheads involved in integrating diverse components will then be reduced to an absolute minimum. Where sites wish to retain licensing control access can be password-based, or forwarded to a server local to the developer.

 **WP 2: Reusable objects for  NLP tasks: CREOLE 2** 

The modules integrated into GATE are chiefly related to IE. To make the system more useful for a wider spectrum of researchers and developers  we will integrate modules for machine translation (MT), information retrieval (IR) [

Strz97], speech recognition (SR), dialogue processing and corpus linguistics and lexicography. 

MT and IR support are straightforward from the perspective of the TIPSTER architecture, but speech recognition and dialogue are more complex. Dr. Steve Renals [

RMB94, RHR96] of the Sheffield Computer Science Department has agreed to act as an advisor to the project in the areas of architectural requirements for supporting integrated speech and text-based NLP research, and on speech sources for IE. The architecture is based on an underlying model of a sequential information source � currently text � with non-sequential (e.g. graph-structured) information associated with it. This will make extension of the current TIPSTER model of a text-based information source (which is indexed by byte offset) to an audio-based information source (which is indexed by time) reasonably straightforward. Having done this we can represent phone or word lattice structures from the speech recognisers in a fashion very similar to the HTK representation standard. To validate this work and provide examples and documentation for others to follow we will integrate the Abbot recogniser [RHR96]. Additional issues in supporting integrated speech work within GATE are: efficiency, and the need for incremental distributed processing as evidenced by e.g. the Verbmobil ICE project (see below). GATE support for dialogue processing means support for documents comprised of dialogue turns, and interface tools for managing user input collection and system output presentation. We will make available the ViewGen system [BW91, LW97, LW96] to exemplify the new facilities. 

Corpus linguistics and lexicography tools such as [

Chr94, MF97] will be added to GATE, based on an analysis of features available in current systems and we will continue work already done with Dr Tony McEnery of Lancaster University on integrating some of his group�s corpus processing tools. The needs of grammar developers for tools such as those presented in [ELNP97] will be analysed, though it seems likely that the best approach is to provide examples of integrating grammars and parsers/generators from external systems, rather than duplicating previous work. We will continue to support data interchange with text markup and learning systems such as [DAH97]. 

 **WP 3: A new model of LE resource use and distribution** 

One of the most interesting developments in CREOLE will be extension of the currently process-oriented resources set to include predominantly data resources such as lexicons, ontologies and thesauri. (This package will make use of the extensions to the GATE core described in WP1.3 above.) Much progress has been made over the last decade in provision of large-scale resources of this type [

WGS95], but despite various standards initiatives, there are still barriers to data resource reuse: 


- each resource has its own representation syntax and corresponding access mode: relational databases in the case of CELEX, a custom C API or Prolog representation in Wordnet, SGML in the case of the British National Corpus;

- resources must be installed locally to be usable (and precisely how this happens, what operating systems are supported etc., varies from site to site).

The consequences of the first bullet are that although linguistic resources normally share some structure in common (e.g. at the most obvious level, lexicons are organised around words and word strings) this commonality is wasted when using a new resource, since the developer has to learn everything afresh each time, and  work which seeks to investigate or exploit commonalities between resources (e.g. to link several lexicons to an ontology) has first to build a layer of access routines on top of each resource. So, for example, if we wish to do task-based evaluation of lexicons by measuring the relative performance of an information extraction system with different instantiations of the lexical resource, we might end up writing code to translate several different resources into SQL or SGML. The consequence of the second bullet above is that there is no way to "try before you buy": no way to examine a data resource for its suitability for your needs before licensing it. Correspondingly, there is no way for a resource provider to give limited access to their products for advertising purposes, nor gain revenue through piecemeal supply of sections of a resource.

Some would argue that the solution is to map all resources into SGML, but experience using the BNC suggests otherwise: to get the best out of it "you have to be an SGML guru" (Tony McEnery, Lancaster). Using databases would also seem an obvious solution (and one supported by the extensive experience of Sheffield with GATE 1, the TIPSTER architecture committee, and the Lancaster University group), but the relational paradigm is also problematic, requiring the representation of the data in cross-referenced table form. As McEnery has also pointed out, using object-oriented database (OODB) technology should resolve this problem. In an OODB the data is represented in a form very close to its original structure using inheritance and containment. We will develop a common programmatic model of the various resources types, implemented in CORBA IDL and/or Java, along with a distributed server for non-local access, and distribute the code required to map them into this model. This model will for the first time provide the research community with a unified means for accessing linguistic resources. The model will be documented in HTML using JavaDoc,

Rational ROSE and SODA. 

The common model of language data resources we propose would be a set of inheritance hierarchies making up a forest or set of graphs. At the top of the hierarchies would be very general abstractions from resources (e.g. a thesarus groups synonyms); at the leaves would be data items that were specific to individual resources (e.g. WordNet synsets have glosses). Program access would be available at all levels, allowing the developer to select an appropriate level of commonality for each application. Note that, although an exciting element of the work would be to provide algorithms dynamically to link common resources e.g. connecting EuroWordNet to LDOCE, this proposal  is not to develop new, but simply to improve access to existing, resources. Notice, also, that it is a proposal about language data quite separate from, though compatible with, the lexical data compression ideas in recent DATR work [

EG96]. This is NOT in any way a new standards initiative, but a way to build on previous initiatives. The issues of standards is a vexed one: experience with repositories of lexical materials (e.g. the CRL Consortium for Lexical Research 1989-93) suggested that if resources had to have standardised formats, they are not deposited or used. The success of WordNet world-wide is a demonstration of how researcher choice can defy any committee's standards. What we propose here is quite different from projects like SEAL [Eva97, KE95] that attempt to conflate different lexical resources: in what we propose, the resources retain their integrity, or "native"' structure. We propose, via an object oriented methodology, a standardised taxonomy and structure only as an index to the links between lexical objects with the same function in the various resources. 

 **2.3 Technological Basis** 

Most of this proposal can be viewed as an extended requirements analysis and high-level design for a long-term infrastructure for language work. Low-level design would be inappropriate at this stage. However, it would also be foolhardy to embark on this type of software development without an idea of the technological risks involved (see e.g. the section on process in [Fow97] for good arguments for doing technology identification an early stage). This section sketches the key technologies we provisionally plan to use for our implementation.

To support the kinds of usage scenarios outlined at the start of section 2, GATE 2 will: be distributed (programs and data may live on several machines); have a flexible graphical interface that displays a wide range of language-oriented data structures; have an efficient underlying data management substrate that is easily extensible to a wide range of existing databases (probably via the JDBC Java�SQL mapping); 16-bit clean Unicode-enabled; be WWW capable, and probably integrated with popular word processing tools like MSWord. To meet these key requirements, GATE 2 will be implemented primarily in the Java language (

java.sun.com). Distributed computing will be implemented using Java RMI (Remote Method Invocation), which is a light-weight alternative to the complexities of CORBA (for more discussion of distribution in NLP see [Zaj97b]). The new Java Beans component model will be used to achieve flexibility � developers will be able to recombine our components for other applications as they wish � and this will also, via MSJ++, give us access to the COM data sharing model underlying WinWord�s new integration mechanisms. The interface will be implemented using the mature model-view-controller architecture handed down from e.g. Smalltalk and now available in e.g. Borland JBuilder and JDK1.2. Backwards-compatibility with GATE 1 will be achieved via the Java Native Interface, which we have used to integrate a simple Java document manager in GATE version 1.5. 

This technology is new, but we have run preliminary experiments with each part of the jigsaw, and are confident of the feasibility of this implementation route.

 **3. The Work Programme** 

The following sections list the milestone objectives in each work package. The project plan is of modular design incorporating comprehensive measurement points. The software team at Sheffield (whose members will lead and advise the project) has an established development process based on iterating cycles of design, implementation, deployment and testing. Each item in the lists below represents a use-case or external requirement on the system, and will be the subject of one or more iterations, which will result in delivered components. Thus testing and integration of new subsystems is distributed across the project, with the advantage that progress can be realistically appraised continuously, and remedial action taken against schedule or quality slippage. Small group software engineering is not well catered for by many mainstream development methods, but our group has several person-decades experience in this type of environment, and our method exploits the core of modern thinking on object oriented and explicit process development. The equipment budget includes support software for design, documentation, rapid prototyping and plan maintenance: Rational Rose; Rational SODA; Borland JBuilder; Microsoft Project. Key references: [

Hum97a], [Boo94]**,** [Fow97].

 **WP 1: The GATE2 core

WP 1.1 A distributed Unicode document manager: GDM 2** 

Integration of distributed document manager from CRL.

Retargeting of existing C++, Tcl and Java APIs on new document manager (to ensure backwards compatibility with GATE 1)

Extension of document manager to speech and multi-media

Addition of information retrieval classes to document manager

Extension of SGML input filter to other document types (including HTML, XML, email and RTF).

 **WP 1.2 A standards-based interface: GGI 2** 

Development of new object-centred Unicode interface with editable documents

Conversion of GATE 1 annotation comparison, annotation creation and annotation visualisation tools to new interface

Provision of interface support for dialogue processing

Creation of an applet version of the interface for WWW-based access to services and resources

 **WP 1.3 Agent-based language processing and a new approach to linguistic resource distribution: GRM** 

Resource manager subsystem with optional autonomous agent control executive

Extension of existing integration automation to distributed CREOLE objects

 **WP 2: CREOLE extension** 

Integration of an existing UK information retrieval system (possibly Okapi)

Integration of other UK NLP components

Integration of the Sheffield/Cambridge Abbot speech recognition system

Integration of the CRL MT system

Integration of ViewGen

Integration of public domain NLP components from e.g. the Stuttgart registry

 **WP 3: Active CREOLE server**  

Deployment of active CREOLE web server (both API-based access and browser access)

Development of linguistic resource object model

Integration of linguistic resources including EuroWordnet, WordNet, CELEX, the ANLT lexicon, CRL LDB and Microkosmos.

Population of CREOLE server with modules and resource

Web demonstrator for IE systems

 **4. Additional Issues** 
 **4.1 Scientific/Technological Relevance** 


Each work package proposed makes advances in Language Engineering (LE) technology and facilitates further advances by the UK R&D Community. The GATE2 NLP architecture is itself novel, and we hope the best available world-wide; the integrated modules we provide will be a rich and novel platform for experimental and technological advance throughout the community, including our own (Sheffield) programme in LE and computational linguistics.

 **4.2 Relevance to Beneficiaries** 

The value of GATE1 to a wide range of R&D groups has already been demonstrated, and GATE2 will advance that standard, helping keep UK work in the front line without the need for direct participation in US competitions, useful as that is. GATE2 will assist system developers both in research and in any branch of the language industries (especially electronic publishing and news dissemination), speeding the integration of functionalities and their testing. This will be evaluated and tested with our collaborators (see attached letters of support).

 **4.3 Dissemination and Exploitation** 

Apart from the standard academic dissemination channels of conferences and journal/book publication (including web and archive publication), we shall continue with specialised workshops, both those we shall organise ourselves, and those we have in the past been invited to join, such as DTI/EPSRC sponsored industrial forums and the Unicom seminar series for industry. GATE is already widely disseminated, and it seems likely at the time of writing that US DARPA may make it an interim NLP architecture standard, which would entrench its R&D position world wide; it has also been used as the basis of a range of EU language engineering projects. We shall continue to publicise it, though without offering maintenance, and will set up a user group and some form of industrially orientated workshop/forum for GATE users, and those interested, during the first year of the project. We have considerable experience in organising workshops of this sort. For both GATE and the IE system, we shall maintain our direct links with US and EU standards-setting bodies, continuing to argue that standards are vital but must be based on systems that are widely acceptable to users. We also participate in ELSE, the EU NLP evolution standards project, and hope to make GATE2 the platform for Europe-wide evaluation in NLP, thus linking EU and US activities to the overall benefit of the UK position as a fulcrum in the field.

We have a developed set of web pages advertising aspects of GATE and our IE work, through our department and through ILASH and we also maintain the linked CLUK and ICCL web sites. We also produce industrially-orientated publicity materials, and receive industrial solicitations as a result of these (and actively solicit others from relevant firms). With them we seek to spread a culture of adapting/customising LE systems to particular industrial requirements. A key feature of the current proposal is that we shall set up a publicly available WWW demo of GATE 2, with as many modalities as proves feasible.

 **4.4 Management** 

The project will be managed by a team led by Yorick Wilks as principal investigator: Rob Gaizauskas will manage requirements capture and Hamish Cunningham system development. Wilks has managed large NLP projects for UK and US agencies, and NLP architecture projects since the beginning of the US TIPSTER project; he was the PI of GATE1. Gaizauskas is widely experienced from the Sussex POETIC project and has designed most of the EPSRC-supported LaSIE system. Cunningham has gained extensive experience with the implementation and deployment of GATE1 and its use in major EU projects. The Sheffield ILASH/CS NLP group has a tight and experienced management structure with its own research coordinator, systems administrator and secretary who ensure delivery of modules, conformance to reporting and delivery deadlines, the archiving of software and the organisation of workshops and related events. The NLP group maintains a seminar series and a set of linked study groups to keep abreast of the literature and to train its research students. The group maintains its own monthly newsletter that also sets out deadlines and milestones: each research project also has its own weekly meeting reporting to the NLP group weekly meeting. We hold regular and frequent meetings with industrial collaborators, whom we also invite to join our departmental Industrial Associates Group.

 **4.5 Manpower and Equipment** 

The proposal requests two RAs: Hamish Cunningham, with substantial engineering experience to lead GATE2 development and module integration, and one junior RA to program on GATE2. Each of these would be fully committed. Each would require a state of the art workstation, since we have no other source for these (our industrial collaborators not being hardware manufacturers), and we also need hardware to support the Active CREOLE Server.

 **4.6 Travel** 

The travel budget represents normal participation at a subset of major conferences to disseminate results (IJCAI, ECAI, AISB, ACL, COLING) with some provision for participation in DARPA events (like MUC) since they are essential if we are to remain a focal part of the international community by participating in the DARPA steering committee.

 **5. References

Adv96**  Advanced Research Projects Agency. *Proceedings of the TIPSTER Text Program (Phase II)*. Morgan Kaufmann, California, 1996. 
 **AHG97**   S. Azzam, K. Humphreys, R. Gaizauskas, H. Cunningham, and Y. Wilks. A Design for Multilingual Information Extraction (poster). In *IJCAI-97*, 1997. 
 **BMSW97**   D. Bikel, S. Miller, R. Schwartz, and R. Weischedel. Nymble: a High-Performance Learning Name-finder. In *Proceedings of the Fifth conference on Applied Natural Language Processing*, 1997. 
 **Boo94**  G. Booch. Object-Oriented Analysis and Design 2nd Edtn. Benjamin/Cummings, 1994.
 **Bri95**   E. Brill. Transformation-Based Error-Driven Learning and Natural Language. *Computational Linguistics*, 21(4), December 1995. 
 **BW91**   A. Ballim and Y. Wilks. *Artificial Believers*. Lawrence Erlbaum Associates, Hillsdale, New Jersey, 1991. 
 **Chr94**   O. Christ. A Modular and Flexible Architecture for an Integrated Corpus Query System. In *Proceedings of the 3rd Conference on Computational Lexicography and Text Research*, 1994. 
 **CFB94** H. Cunningham, M. Freeman, and W.J. Black. Software Reuse, Object-Oriented Frameworks and Natural Language Processing. In *Proceedings of the conference on New Methods in Natural Language Processing (NeMLaP-1)*, Manchester, 1994. 

 **CGW95**   H. Cunningham, R. Gaizauskas, and Y. Wilks. A General Architecture for Text Engineering (GATE) � a new approach to Language Engineering R&D. Technical Report CS � 95 � 21, Department of Computer Science, University of Sheffield, 1995. Also available as http://xxx.lanl.gov/ps/cmp-lg/9601009. 
 **CHGW96**   H. Cunningham, K. Humphreys, R. Gaizauskas, and Y. Wilks. TIPSTER-Compatible Projects at Sheffield. In *Advances in Text Processing, TIPSTER Program Phase II*. DARPA, Morgan Kaufmann, California, 1996. 
 **CHGW97a**   H. Cunningham, K. Humphreys, R. Gaizauskas, and Y. Wilks. GATE � a TIPSTER-based General Architecture for Text Engineering. In *Proceedings of the TIPSTER Text Program (Phase III) 6 Month Workshop*. DARPA, Morgan Kaufmann, California, May 1997. 
 **CHGW97b**   H. Cunningham, K. Humphreys, R. Gaizauskas, and Y. Wilks. Software Infrastructure for Natural Language Processing. In *Proceedings of the Fifth Conference on Applied Natural Language Processing (ANLP-97)*, March 1997. Available as http://xxx.lanl.gov/ps/9702005. 
 **Cun94**  H. Cunningham, M. Freeman, and W.J. Black. Software Reuse, Object-Oriented Frameworks and Natural Language Processing. In *Proceedings of the conference on New Methods in Natural Language Processing (NeMLaP-1)*, Manchester, 1994.
 **CWG96a**   H. Cunningham, Y. Wilks, and R. Gaizauskas. GATE � a General Architecture for Text Engineering. In *Proceedings of the 16th Conference on Computational Linguistics (COLING-96)*, Copenhagen, August 1996. 
 **CWG96b**   H. Cunningham, Y. Wilks, and R. Gaizauskas. Software Infrastructure for Language Engineering. In *Proceedings of the AISB Workshop on Language Engineering for Document Analysis and Recognition*, Brighton, U.K., April 1996. 
 **CWG96c**   H. Cunningham, Y. Wilks, and R. Gaizauskas. New Methods, Current Trends and Software Infrastructure for NLP. In *Proceedings of the Conference on New Methods in Natural Language Processing (NeMLaP-2)*, Bilkent University, Turkey, September 1996. Also available as http://xxx.lanl.gov/ps/cmp-lg/9607025. 
 **DAH97**   D. Day, J. Aberdeen, L. Hirschman, R. Kozierok, P. Robinson, and M. Vilain. Mixed-Initiative Development of Language Processing Systems. In *Proceedings of the 5th Conference on Applied NLP Systems (ANLP-97)*, 1997. 
 **EG96 Evans, R. and Gazdar, G. DATR: A language for lexical knowledge representation. In *Computational Linguistics*, volume 22, 1996.**  **ELNP97**   D. Estival, A. Lavelli, K. Netter, and F. Pianesi, editors. *Computational Environments for Grammar Development and Linguistic Engineering*. Association for Computational Linguistics, July 1997. Madrid, ACL-EACL'97. 
 **Fow97** M. Fowler. UML Distilled: Applying the Standard Modelling Language. Addison-Wesley, 1997.
 **Eva97**  R. Evans. SEAL: Structural Enhancement of Automatically-acquired Lexicons. http://www.itri.brighton.ac.uk/projects/seal. 1997
 **GHAW97**  R. Gaizauskas, K. Humphreys, S. Azzam, and Y. Wilks. Concepticons vs. Lexicons: An Architecture for Multilingual Information Extraction. In M.T. Pazienza, editor, *Proceedings of the Summer School on Information Extraction (SCIE-97)*, pages 28�43. Springer-Verlag, 1997.
 **GCW96**   R. Gaizauskas, H. Cunningham, Y. Wilks, P. Rodgers, and K. Humphreys. GATE � an Environment to Support Research and Development in Natural Language Engineering. In *Proceedings of the 8th IEEE International Conference on Tools with Artificial Intelligence (ICTAI-96)*, Toulouse, France, October 1996. 
 **GH96**   R. Gaizauskas and K. Humphreys. Using verb semantic role information to extend partial parses via a co-reference mechanism. In J. Carroll, editor, *Proceedings of the Workshop on Robust Parsing*, pages 103�113, Prague, Czech Republic, August 1996. European Summer School in Language, Logic and Information. 
 **GH97a**   R. Gaizauskas and K. Humphreys. Quantitative Evaluation of Coreference Algorithms in an Information Extraction System. In S. Botley and T. McEnery, editors, *Discourse Anaphora and Anaphor Resolution*. In Press. Also available as CS � 97 � 19.
 **GH97b** R. Gaizauskas and K. Humphreys. Easing a semantic network for information extraction. *Journal of Natural Language Engineering*. In Press
 **Got98**  Y. Gotoh, S, Renals, R. Gaizauskas, G. Williams, and H. Cunningham. Named Entity Tagged Language Models for LVCSR. Technical Report CS-98-05, Department of Computer Science, University of Sheffield, 1998.
 **GR97a**   R. Gaizauskas and A.M. Robertson. Coupling information retrieval and information extraction: A new text technology for gathering information from the web. In *Proceedings of RIAO'97*, Montreal, 1997. 
 **GR97b**   R. Gaizauskas and P. Rodgers. NL Module Evaluation in GATE. In *Proceedings of the SALT club workshop on Evaluation in Speech and Language Technology*, 1997. 
 **Gri96**   R. Grishman. TIPSTER Architecture Design Document Version 2.2. Technical report, DARPA, 1996. Available at http://www.tipster.org/. 
 **Gri97**   R. Grishman. Information Extraction: Techniques and Challenges. In *Information Extraction: a Multidisciplinary Approach to an Emerging Information Technology*, Springer 1997. 
 **GS96**   R. Grishman and B. Sundheim. Message understanding conference - 6: A brief history. In *Proceedings of the 16th International Conference on Computational Linguistics*, Copenhagen, June 1996. 
 **GW97 R. Gaizauskas, and Y. Wilks. Information Extraction: Beyond Document Retrieval. In *Journal of Documentation*, 1996.**  **GWH95**   R. Gaizauskas, T. Wakao, K Humphreys, H. Cunningham, and Y. Wilks. Description of the LaSIE system as used for MUC-6. In *Proceedings of the Sixth Message Understanding Conference (MUC-6)*. Morgan Kaufmann, California, 1995. 
 **Har91**   P. Harrison. Evaluating Syntax Performance of Parsers/Grammars of English. In *Proceedings of the Workshop on Evaluating Natural Language Processing Systems, ACL*, 1991. 
 **Hum97a**  W.S. Humphreys. Introduction to the Personal Software Process. Addison-Wesley, 1997.
 **Kam97**   M. Kameyama. Information Extraction across Linguistic Boundaries. In *AAAI Spring Symposium on Cross-Language Text and Speech Processing*, Stanford University, 1997.
 **KE95**  Kilgarriff, A. and Evans, R., MRDs, Standards and How to Do Lexical Research Proceedings of the Language Engineering Conference, London, October 1995. 
 **KGW94** A. Krotov, R. Gaizauskas and Y. Wilks. Acquiring a stochastic context-free grammar from the penn treebank. In *Proceedings of the Third International Conference of the Cognitive Science of Natural Language Processing (CSNLP94)*, pages 79�86, Dublin, July 1994. 
 **Lee95** M. Lee. A goal ascription based approach to conversational implicature. In *Proceedings of the 14th Workshop of the UK Planning and Scheduling Special Interest Group*, Essex, 1995. 

 **LCB+97** D. Levy, R. Catizone, B. Battacharia, A. Krotov and Y. Wilks. CONVERSE: A Conversational Companion. In *Proceedings of the 1^st^ International Workshop on Human-Computer Conversation.* 1997
 **LW96**   M. Lee, Y. Wilks. An ascription-based approach to speech acts. *Proceedings of the 16th Conference on Computational Linguistics (COLING-96)*, Copenhagen, 1996. 
 **LW96a** M. Lee and Y. Wilks. An ascription-based approach to speech acts. In *Proceedings of the 16th Conference on Computational Linguistics (COLING-96)*, Copenhagen, 1996. 
 **LW96b** M. Lee and Y. Wilks. Speech acts on demand. In *Proceedings of the 5th International Pragmatics Conference*, Mexico City, 1996. 

 **LW97**   M. Lee, Y. Wilks. Eliminating deceptions and mistaken belief to infer conversational implicature. In *IJCAI-97 workshop on Conflict, Co-operation and Collaboration in Dialogue Systems*, Tokyo, 1997. 
 **MBT97**   D. McKelvie, C. Brew, and H. Thompson. Using SGML as a Basis for Data-Intensive NLP. In *Proceedings of the fifth Conference on Applied Natural Language Processing (ANLP-97)*, Washington, DC, 1997. 
 **MF97**   A. Mikheev, S. Finch. A Workshop for Finding Structure in Text. In *Fifth Conference on Applied NLP (ANLP-97)*, Washington, DC, 1997. 
 **Mor96**   R.G. Morgan. An architecture for user defined information extraction. Technical Report 8/96, dept. Computer Science, University of Durham, 1996. 
 **RGHC97**   P.J. Rodgers, R. Gaizauskas, K. Humphreys, and H. Cunningham. Visual Execution and Data Visualisation in Natural Language Processing. In *IEEE Visual Language, Capri, Italy*, 1997. 
 **RHR96**   T. Robinson, M. Hochberg, and S. Renals. The use of recurrent networks in continuous speech recognition. In C. H. Lee, K. K. Paliwal, and F. K. Soong, editors, *Automatic Speech and Speaker Recognition � Advanced Topics*, chapter 10, pages 233�258. Kluwer Academic Publishers, Amsterdam, 1996. 
 **RMB94**   S. Renals, N. Morgan, H. Bourlard, M. Cohen, and H. Franco. Connectionist probability estimators in HMM speech recognition. *IEEE Transactions on Speech and Audio Processing*, 2:161�175, 1994. 
 **Sch94**   J. Schütz. Developing Lingware in ALEP. *ALEP User Group News, CEC Luxembourg*, 1(1), October 1994. 
 **Sim94**   N. K. Simkins. An Open Architecture for Language Engineering. In *First CEC Language Engineering Convention, Paris*, 1994. 
 **Strz97 T., Strzalkowski, F., Lin, J., Wang, and J. Perez-Caballo. Natural Language Information Retrieval. Chapter Evaluating Natural Language Processing Techniques in Information Retrieval, pages 113�146. Kluwer Academic Publishers, 1997.**  **Thu97**   G. Thurmair. Information extraction for intelligence systems. In *Natural Language Processing: Extracting Information for Business Needs*, pages 135�149, London, March 1997. Unicom Seminars Ltd. 
 **TM96**   H. Thompson and D. McKelvie. A Software Architecture for Simple, Efficient SGML Applications. In *Proceedings of SGML Europe '96, Munich*, 1996. 
 **TWY96**   Y. Takemoto, T. Wakao, H. Yamada, R. Gaizauskas, and Y. Wilks. Description of the NEC/Sheffield System Used for MET Japanese. In *Proceedings of the TIPSTER Phase II Workshop*, 1996. 
 **VD96**   M. Vilain and D. Day. Finite-state phrase parsing by rule sequences. In *Proceedings of COLING-96*, 1996. 
 **WB96** Y. Wilks and T. By. Procedural conventions for reference resolution in belief environments. Submitted to *Cognitive Science*, 1998. 
 **WBB91** Y. Wilks, J. Barnden, and A. Ballim. Belief ascription, metaphor and intensional identity. *Cognitive Science*, 15(1), 1991. 
 **WBW91** Y. Wilks, J. Barnden, and J. Wang. Your metaphor or mine: Belief ascription and metaphor interpretation. In *Proceedings of IJCAI-91*, Sydney, Australia, 1991. 

 **WG97**   Y. Wilks and R. Gaizauskas. LaSIE jumps the GATE. In *Natural Language Processing: Extracting Information for Business Needs*, UNICOM, 1997. 
 **WGS95**   Y. Wilks, L. Guthrie, and B. Slator. *Electric Words: Lexicons, Dictionaries and Meanings*. MIT Press, Cambridge, MA, 1995. 
 **WGW96**   T. Wakao, R. Gaizauskas, and Y. Wilks. Evaluation of an algorithm for the recognition and classification of proper names. In *Proceedings of the 16th International Conference on Computational Linguistics (COLING96)*, pages 418�423, Copenhagen, 1996. 
 **Wil96** Y. Wilks. Senses and texts. *Computers and Humanities, 1997.* 
 **Zaj97b**   R. Zajac. An Open Distributed Architecture for Reuse and Integration of Heterogeneous NLP Components. In *Proceedings of the 5th conference on Applied Natural Language Processing (ANLP-97)*, 1997.


---

Back to my home page
